# *Panax notoginseng* saponins alleviate skeletal muscle insulin resistance by regulating the IRS1–PI3K–AKT signaling pathway and GLUT4 expression

**DOI:** 10.1002/2211-5463.12635

**Published:** 2019-04-26

**Authors:** Xuan Guo, Wen Sun, Guangbin Luo, Lili Wu, Guangyuan Xu, Dan Hou, Yi Hou, Xiangyu Guo, Xiaohong Mu, Lingling Qin, Tonghua Liu

**Affiliations:** ^1^ Dongfang Hospital of Beijing University of Chinese Medicine China; ^2^ Key Laboratory of Health Cultivation of the Ministry of Education Beijing University of Chinese Medicine China; ^3^ Beijing Key Laboratory of Health Cultivation Beijing University of Chinese Medicine China; ^4^ School of Life Sciences Beijing University of Chinese Medicine China; ^5^ Department of Traditional Chinese Medicine Fu Xing Hospital of Capital Medical University Beijing China; ^6^ Department of Orthopaedics Dongzhimen Hospital of Beijing University of Chinese Medicine China; ^7^ Science and Technology Department Beijing University of Chinese Medicine China

**Keywords:** diabetes, GLUT4, IRS1–PI3K–AKT signaling, KKAy mice, *Panax notoginseng* saponins, skeletal muscle

## Abstract

*Panax notoginseng* saponins (PNS) are a commonly used traditional medicine to treat diabetes in China. Recent studies have confirmed their anti‐diabetic effects, but the underlying mechanisms have remained unclear. The present study was designed to explore whether PNS decrease hyperglycemia by improving insulin sensitivity in skeletal muscle and to elucidate the molecular mechanisms. The anti‐diabetic effects of PNS were analyzed in a skeletal myoblast cell line, C2C12, and in high fat diet‐induced diabetic KKAy mice. C2C12 cells were treated with PNS (50, 100, and 200 μg·L^−1^) and examined for glucose uptake, cell viability and expression of components of the phosphoinositide 3‐kinase (PI3K)–protein kinase B (AKT) signaling pathway. KKAy mice were intraperitoneally injected with PNS (200 mg·kg^−1^) for 6 weeks. Body weight, blood glucose, serum insulin, serum lipid, glucose and insulin tolerance were measured to evaluate the anti‐diabetic effects of PNS. Pathological changes, apoptosis and the PI3K–AKT signaling pathway were analyzed in KKAy skeletal muscle. PNS significantly increased insulin‐induced glucose uptake, but did not affect the cell viability of C2C12 cells. In addition, PNS reduced blood glucose and serum insulin levels and improved glucose tolerance and insulin tolerance of KKAy mice. Pathological changes and apoptosis of skeletal muscle were relieved by PNS treatment. Moreover, PNS treatment enhanced expression of mRNA encoding IRS1 and GLUT4, as well as the protein expression of phosphorylated (p) ‐insulin receptor substrate 1 (IRS1), p‐PI3K, p‐AKT and glucose transporter type 4 (GLUT4) in C2C12 and KKAy mouse muscle. Collectively, these data indicate that PNS reduces hyperglycemia and insulin resistance through up‐regulating GLUT4 expression and the IRS1–PI3K–AKT signaling pathway. Furthermore, PNS alleviated diabetes skeletal muscle pathological damage. Thus, our data suggest that PNS may be promising anti‐diabetic compounds.

**Abbreviations**
2‐DG: 2‐deoxyglucose
AKT: protein kinase B
AUC: area under the curve
DM: diabetes mellitus
FBG: fasting blood glucose
Fins: fasting serum insulin
GLUT4: glucose transporter type 4
HDL: high‐density lipoprotein
HE: hematoxylin and eosin
HOMA‐IR: homeostasis model assessment of insulin resistance
IRS: insulin receptor substrate
IRS1: insulin receptor substrate 1
ITT: insulin tolerance test
LDL: low‐density lipoprotein
OGTT: oral glucose tolerance test
PI3K: phosphoinositide 3‐kinase
PNS: *Panax notoginseng* saponins
RBG: random blood glucose
TC: total cholesterol
TG: triglycerides
TUNEL: terminal deoxynucleotidyl transferase dUTP nick end labeling

Diabetes mellitus (DM) is a common metabolic disorder characterized by abnormally high blood glucose levels, which can cause multi‐systemic complications, including diabetic ketoacidosis, kidney failure, cardiovascular damage, and even death 1. Global DM prevalence is estimated to reach 552 million by 2030 2. Ninety percent of DM cases are categorized as type 2 DM 3, 4. Insulin resistance is the primary cause of type 2 DM and refers to individuals whose target cells lose their sensitivity to insulin. Insulin resistance causes abnormal glucose tolerance, arterial hypertension, and glucose and lipid metabolism disorders, which eventually lead to multiple complications such as non‐alcoholic fatty liver disease, cardiovascular disease and metabolic disorders 5, 6, 7. Therefore, improving insulin resistance has become the primary strategy for treating DM.

Skeletal muscle is a major reservoir for postprandial glucose storage that contributes to peripheral insulin resistance in DM. Energy consumption in skeletal muscle accounts for more than 30% of that of the whole body, and 80% of blood glucose is absorbed by skeletal muscles. As an insulin target, muscle cells are important sites of energy balance, consumption and storage. Therefore, improving insulin resistance in skeletal muscle has been an effective strategy in diabetes drug development 3, 4.

*Panax notoginseng* is a source of traditional Chinese medicine that has been used to treat cardiovascular disease and diabetes for thousands of years in China 8. *Panax notoginseng* saponins (PNS) are the major active ingredients in *Panax notoginseng*. Several studies have shown that PNS lower blood glucose and lipid levels 9, 10. PNS treatment was observed to significantly increased cell viability, intracellular superoxide dismutase and catalase and decrease reactive oxygen species and malondialdehyde in rat retinal capillary endothelial cells exposed to high glucose 11. The diabetes‐induced oxidative stress was attenuated and low active protein kinase B (AKT) expression was restored in corpora cavernosa by PNS treatment 12. Therefore, therapeutic considerations of PNS have focused on their anti‐oxidative effect. Kim *et al*. 10 reported that PNS increase glucose uptake through up‐regulating membrane glucose transporter type 4 (GLUT4) in adipocytes. However, the mechanisms of PNS treatment of diabetes still need further exploration. Since muscle is a major organ for treating insulin resistance and also expresses GLUT4 as well as AKT as key factors in glucose metabolism, we investigated the effects of PNS on glucose metabolism and uptake in skeletal muscle and explore related molecular mechanisms.

GLUT4 is an insulin‐regulated glucose transporter normally found in intracellular vesicles in fat and muscle cells under low insulin conditions. However, high levels of insulin can induce plasma membrane translocation of GLUT4 from intracellular vesicles as a means of increasing cellular glucose uptake 13, 14. Phosphoinositide 3‐kinase (PI3K) plays a vital role in insulin‐induced glucose uptake signaling in skeletal muscle. PI3K is up‐regulated by insulin receptor substrate (IRS), which binds and activates its downstream effector, AKT, to cause GLUT4 translocation to the membrane. Alterations in GLUT4 translocation cause glucose uptake disorders, resulting in insulin resistance 15. However, whether PNS alleviate insulin resistance through these signaling pathways has remained unclear.

Therefore, the current study aimed to investigate whether PNS could decrease insulin resistance of skeletal muscle and explore the molecular mechanisms. We hypothesized that PNS could regulate insulin resistance in skeletal muscle through activation of the PI3K–AKT pathway and GLUT4 expression. Thus, experiments were carried out in a mouse myoblast cell line, C2C12, and in the high fat diet‐induced spontaneous type 2 diabetes KKAy mouse model. The effect of PNS on PI3K–AKT signaling and GLUT4 expression was further explored.

## Materials and methods

### *Panax notoginseng* saponins

*Panax notoginseng* saponins with Chinese drug reference standard were purchased from the National Institutes for Food and Drug Control of China (Batch lot: 110870‐201002; Beijing, China). This product is a total saponin made from the main root or rhizome of *Panax notoginseng* (Burk) F.H. Chen. It contains notoginsenoside R1 (6.9%), ginsenoside Rg1 (28.0%), ginsenoside Re (3.8%), ginsenoside Rb1 (29.7%) and ginsenoside Rd (7.3%).

### Cell culture

C2C12 cells, purchased from Creative Bioarray (Shirley, NY, USA), were cultured in complete Dulbecco's modified Eagle's medium (DMEM, 1 g·L^−1^ d‐glucose) (Gibco, New York, NY, USA) containing 10% FBS (Hyclone, Omaha, NE, USA) and 1% penicillin–streptomycin (Gibco) at 37 °C with 5% CO_2_. FBS in the culture medium was replaced with 2% horse serum (Hyclone) once the cells reached 90–95% confluence. Cells were incubated for 5–7 days and the cells spontaneously differentiated and fused to form myotubes that were used as negative control (N group). Cells in the high glucose group (C group) and PNS group were cultured in high‐glucose DMEM (4.5 g·L^−1^ d‐glucose; Gibco), other conditions were the same as described above. For cellular synchronization, the myotubes were cultured overnight in DMEM with 1% penicillin–streptomycin without serum. The cells were washed with pre‐warmed PBS three times and then incubated with PNS (50, 100, and 200 μg·mL^−1^) in DMEM (1 g·L^−1^ d‐glucose) without FBS for 12 h. These cells were then used for functional analysis.

### Cell viability assay

Cell viability was measured using Cell Counting Kit‐8 (CCK‐8) (DOJN, Tokyo, Japan). C2C12 cells were seeded at 1 × 10^4^ per well on a 96‐well culture plate and incubated in 100 μL complete medium (as described above) for 24 h. About 10 μL PNS in DMEM was applied to the corresponding wells at final concentrations of 0, 50, 100, and 200 μg·mL^−1^ and incubated for 24 h. On the following day, 10 μL of CCK‐8 solution was added to each well and incubated for another 2 h. The absorbance was recorded at 450 nm. Each assay condition included 10 samples. Cell viability was calculated as: PNS medium absorbance/control group absorbance.

### Glucose uptake assay

C2C12 cells were seeded at 5000 cells/well/100 μL in a 96‐well culture plate with complete DMEM for 3 days. Cell differentiation was induced by horse serum for 2 days. Glucose uptake of PNS‐treated C2C12 cells was assessed using the Screen Quest™ Fluorimetric Glucose Uptake Assay Kit (AAT Bioquest, CA, USA) following the manufacturer's instructions. About 50 μL of the 2‐deoxyglucose (2‐DG) uptake assay mixture was detected using a FLUOstar Omega microplate reader (BMG LABTECH, Offenburg, Germany) at *E*_x_/*E*_m_ = 544/590 nm.

### Animals

Seven‐week‐old KKAy and C57BL/6J mice (Institute of Medical Laboratory Animals, Chinese Academy of Medical Sciences, Beijing) were singly housed in the Beijing Experimental Animal Research Center according to a protocol approved by the Beijing University of Chinese Medicine Animal Care Committee. C57BL/6J mice were fed a normal chow diet (HFK Bioscience Co., Beijing, China) under 55 ± 10% humidity and 12 h/12 h light/darkness with free access to drinking water at 23 ± 2 °C. KKAy mice were fed a high fat diet (78.8% basal diet, 10% egg yolk powder, 10% lard, 1.0% cholesterol, 0.2% bile salt; Chinese Academy of Medical Sciences Laboratory Animal Research Institute).

After 2 weeks of adaptive feeding, 16 KKAy mice with random blood glucose (RBG) > 13.9 mmol·L^−1^ over a period of at least 3 days were collected and randomly divided into two groups: the PNS group and the KKAy group (KK group). Randomized male C57BL/6J mice were used as the normal group (C57 group). PNS (200 mg·kg^−1^ body weight) was intraperitoneally injected into the mice in the PNS group for 6 weeks at 07.00 h each morning. The same volume and schedule was used for the normal saline injections in the KK and C57 groups.

Body weight and fasting blood glucose (FBG) was measured 12 h after fasting and RBG was detected at 07.00 h every 2 weeks.

### Mouse blood glucose, oral glucose tolerance test and insulin tolerance test

Blood glucose was measured using the glucose dehydrogenase method with the Glucose Oxidase kit (Applygen Technologies Inc., Beijing, China). Briefly, the sample was mixed with the working solution and incubated at 37 °C for 20 min. FBG was calculated using an absorbance reading at 550 nm.

The oral glucose tolerance test (OGTT) and insulin tolerance test (ITT) experiments were performed in each group of mice at week 6. For OGTT, mice were fasted overnight and then orally administrated 2 g·kg^−1^ glucose. FBG was measured at time 0 (before glucose administration), 30, 60 and 120 min. The area under the curve (AUC) of the OGTT was calculated. For ITT analysis, mice were fasted without access to water for 4 h, and then subcutaneously injected with 1 U·kg^−1^ insulin (Jiangsu Wanbang Biochemistry Medicine Company, Xuzhou, China). FBG was measured at time 0 (before injection), 30, 60, 90 and 120 min. The AUC of the ITT was then calculated.

### Homeostasis model assessment of insulin resistance

Fasting serum insulin (Fins) was analyzed using the Mouse Ultrasensitive Insulin ELISA kit (ALPCO, Salem, NH, USA). Briefly, the samples in a 96‐well plate were incubated with 10 μL of insulin antibody and 50 μL of streptavidin–horseradish peroxidase (from the kit) at 37 °C for 60 min. The coloring reagent was added to the samples in the dark for 10 min at 37 °C. Finally, the stop reagent was added and the absorbance at 450 nm was detected. The insulin resistance index was calculated as: Homeostasis Model Assessment of Insulin Resistance (HOMA‐IR) = FBG (mmol·L^−1^) × Fins (mIU) L^−1^/22.5.

### Blood lipid assay

Serum levels of high density lipoprotein (HDL) cholesterol (C), low density lipoprotein (LDL)‐C, total cholesterol (TC), and triglycerides (TG) were measured using HDL‐C, LDL‐C, TC, and TG Kits (Zhong Sheng Bei Kong, Beijing, China) following the manufacturer's instructions.

### Histology analysis

Skeletal muscle was isolated from mice after they were sacrificed. Muscles were immediately fixed in 4% paraformaldehyde for 24 h, embedded in paraffin, and then sliced into 5 μm‐thick sections for hematoxylin and eosin (HE) staining.

### Electron microscopy analysis

For electron microscopy analysis, each muscle sample was cut into 1 × 1 × 1 mm pieces, fixed in 2.5% glutaraldehyde for 2 h and immobilized with 2% osmium acid for 1.5 h. Then the muscle was dehydrated with acetone, soaked in embedding agent at 37 °C overnight, and sectioned with an ultrathin‐slicing machine and placed on copper mesh grids. Sections were stained with uranyl acetate and citric acid and observed using transmission electron microscopy (Beijing University of Traditional Chinese Medicine Research Center).

### TUNEL assay

The anti‐apoptosis properties of PNS were measured using a terminal deoxynucleotidyl transferase dUTP nick end labeling (TUNEL) assay kit (Roche Diagnostics, Basel, Switzerland). Briefly, the paraffin sections of skeletal muscles were routinely dewaxed, treated with protease K (Merck, Darmstadt, Germany) at room temperature for 30 min, incubated with 50 mL of TUNEL reaction mixture. Apoptotic cells were observed as a brown color by 3,3′‐diaminobenzidine staining.

### Real‐time fluorescence quantitative PCR

Total RNA was isolated from cells and muscle with Trizol regent (SolarBio Life Science, Beijing, China) and treated with DNaseI (Promega, Madison, WI, USA). About 1.5 μg total RNA was reverse‐transcribed into cDNA using GoScript Reverse Transcription Kit (Thermo Fisher Scientific, Waltham, MA, USA). Each 1 μL of the synthesized cDNA was used as a template for real‐time PCR analysis with the GoTaq qPCR kit (Promega) according to the manufacturer's instructions. PCR was performed using the Applied Biosystems 7500 Real‐Time PCR System (Thermo Fisher Scientific) as follow: 94 °C for 15 min; 40 cycles of 94 °C for 15 s, 60 °C for 60 s; and 72 °C for 10 min. The results were analyzed by the relative quantitative ($2^{-\Delta \Delta C_{T}}$) method. The primer sequences were as follows:

GLUT4 forward primer: 5′‐GGTTGGTGCCTATGTATGT‐3′,
reverse primer: 5′‐CGGATGATGTAGAGGTATCG‐3′;
IRS1 forward primer: 5′‐AATAGCCGTGGTGATTACAT‐3′,
reverse primer: 5′‐CAGAAGCAGAAGCAGAGG‐3′;
β‐actin forward primer: 5′‐TGTTGTCCCTGTATGCCTCT‐3′,
reverse primer: 5′‐TAATGTCACGCACGATTTCC‐3′.

### Western blot analysis

Protein concentrations of cells and tissue were measured with the Protein Quantitative Kit (Applygen Technologies Inc.). Protein samples were separated by 10% SDS/PAGE gel and transferred onto a poly(vinylidene difluoride) membrane (Merck Millipore, Bedford, MA, USA) by a semi‐dry transfer (Bio‐Rad, Hercules, CA, USA). After blocking with 5% milk, the membrane was incubated with primary antibodies overnight at 4 °C. The primary antibodies included: anti‐phospho (p)‐PI3Kp85 (1 : 2000, ab182651; Abcam, Cambridge, UK), anti‐p‐Tyr612‐IRS1 (1 : 2000; Thermo Fisher Scientific), anti‐p‐AKT (Ser473) (1 : 2000; CST, 4060 Cell Signaling Technology, Danvers, MA, USA), anti‐AKT (1 : 1000; CST, 4691 Cell Signaling Technology), and anti‐GLUT4 (1 : 1000; ab654 Abcam). After washing, the membrane was incubated with the corresponding peroxidase‐labeled secondary antibodies. Proteins were visualized using a chemiluminescence kit. (ZSGB‐BIO, Beijing, China). β‐Actin was used as an internal reference. The expressed proteins were quantified by densitometry analysis using imagej software (National Institutes of Health, Bethesda, MD, USA).

### Statistical analysis

All tested data were analyzed using spss 17.0 statistical software (SPSS Inc., Chicago, IL, USA). The data are presented as mean ± standard deviation (SD). The difference between groups was analyzed with one‐way ANOVA followed by Tukey's multiple comparison test. *P *<* *0.05 represents a statistically significant difference.

## Results

### PNS do not affect C2C12 cell viability

C2C12 cell viability in response to different PNS concentrations (0, 50, 100, and 200 μg·L^−1^) was measured. As shown in Fig. S1, cell viability was not significantly different among the groups (*P* ˃ 0.05), indicating that PNS do not cause cell toxicity.

### Anti‐diabetic properties of PNS on glucose metabolism in C2C12 cells and mouse skeletal muscle

**Figure 1:**
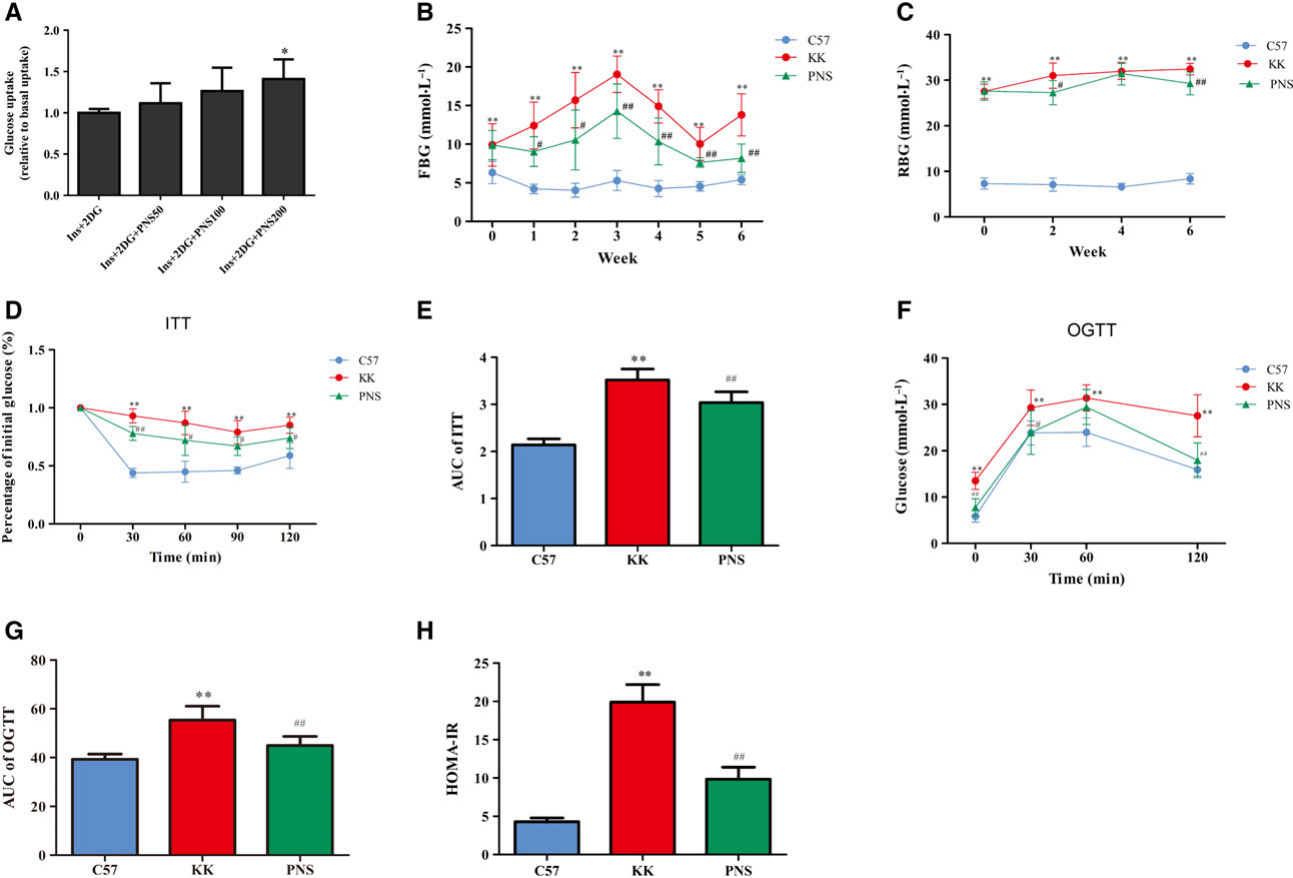
Antidiabetic effect of PNS on C2C12 cells and KKAy mice. (A) Glucose uptake was detected in C2C12 cells treated with PNS and presented in comparison with Insulin (Ins)+2‐DG, which was a standard cellular glucose uptake reference and normalized as 1. *P < 0.05 vs. the insulin+2‐DG group, (*n* = 3). (B,C) Mouse FBG (B) and mouse RBG (C) were evaluated in each group (*n* = 8). (D–G) ITT (D) and glucose tolerance test (OGTT) (F) were determined, and AUC of ITT (E) and OGTT (G) were calculated (*n* = 8). (H) HOMA‐IR was calculated (*n* = 7). The results are expressed as the mean ± SD. *P < 0.05 vs. C57 group, **P < 0.01 vs. C57 group, ^#^ *P* < 0.05 vs. KK group, ^##^ *P* < 0.01 vs. KK group (Tukey's test).

We next investigated the effects of PNS on glucose metabolism. The effect of varying PNS concentrations (50, 100, and 200 μg·L^−1^) on glucose uptake with insulin was measured in C2C12 cells. Insulin+2‐DG only was used as a standard glucose uptake control. We found that 200 μg·L^−1^ PNS induced a maximal response and significantly increased cell glucose uptake in C2C12 cells with insulin compared to the control insulin+2‐DG group (*P* < 0.05) (Fig. 1A).

The effect of PNS on glucose metabolism in mice is shown in Fig. 1B,C. KKAy mice developed a stable higher FBG and RBG than the C57 group. PNS treatment decreased FBG at each time point in the PNS group compared to the KK group. This effect was greater during weeks 3–6 (*P *<* *0.01). For RBG, at week 2 and week 6, PNS attenuated RBG levels in the PNS group compared to the KK group.

Body weight was monitored every 2 weeks. It was significantly increased at each recorded time point in the KK group compared to the C57 group (*P *<* *0.01), but there was no significant decrease of body weight change after PNS treatment (Table 1).

**Table 1:** Influence of PNS on body weight (g). The results are expressed as the mean ± SD

Group	Before PNS	PNS week 2	PNS week 4	PNS week 6
C57	20.23 ± 1.8	22.45 ± 1.13	22.49 ± 1.07	24.06 ± 1.39
KK	32.25 ± 5.28**	33.45 ± 3.7**	37.19 ± 4.03**	37.53 ± 4.54**
PNS	32.49 ± 1.77**	38.31 ± 3.13**	38.65 ± 3.8**	41.1 ± 4.28**

**P <* *0.01 compared to the C57 group at corresponding recording time (Tukey's test, *n* = 8).

### PNS improve insulin resistance and glucose tolerance

An ITT was carried out at week 6. As shown in Fig. 1D,E, there was an impaired insulin sensitivity in the KK group since the blood glucose was significantly elevated at each time point after insulin injection compared to the C57 group. The AUC was also significantly increased in the KK group compared to the C57 group while the AUC of the PNS group was significantly lower than that of the KK group (*P *<* *0.01) (Fig. 1E), indicating PNS treatment could increase the insulin sensitivity.

A OGTT was carried out at week 6 in consideration of glucose intolerance and insulin resistance. As shown in Fig. 1F,G, KKAy mice had impaired glucose tolerance with blood glucose levels significantly higher at each time point and AUC higher than in the C57 group. After PNS treatment, blood glucose and AUC were decreased compared to those of the KK group.

The HOMA‐IR was at least 4 times higher in the KKAy mice compared to normal controls. However, PNS treatment significantly decreased HOMA‐IR (Fig. 1H).

All of the above results indicate that PNS promote glucose metabolism, regulate FBG, RBG, ITT, OGTT and HOMA‐IR profiles, and improve insulin resistance in diabetic mice.

### The effect of PNS on blood lipid levels in the KKAy mouse model

As for mouse blood lipid levels, TG, TC, HDL, and LDL were significantly increased in the KK group compared to the C57 group (*P *<* *0.01). PNS treatment significantly decreased TC and LDL levels (*P *<* *0.01), but no significant differences were observed with TG and HDL levels (Table 2).

**Table 2:** The effect of PNS on mouse blood lipids (mmol·L^−1^). The results are expressed as the mean ± SD

Group	TG	TC	HDL	LDL
C57	0.79 ± 0.11	1.8 ± 0.37	2.14 ± 0.27	0.04 ± 0.09
KK	3.57 ± 1.36**	5.6 ± 1.23**	4.32 ± 0.96**	1.38 ± 0.38**
PNS	2.45 ± 0.88**	3.62 ± 0.67** ^,^ ##	3.45 ± 0.68**	0.77 ± 0.09** ^,^ ##

**P <* *0.01 compared to the C57 group. ^##^ *P *<* *0.01 compared to the KK group (Tukey's test, *n* = 7).

### PNS attenuate pathological damage in KKAy mouse skeletal muscle

**Figure 2:**
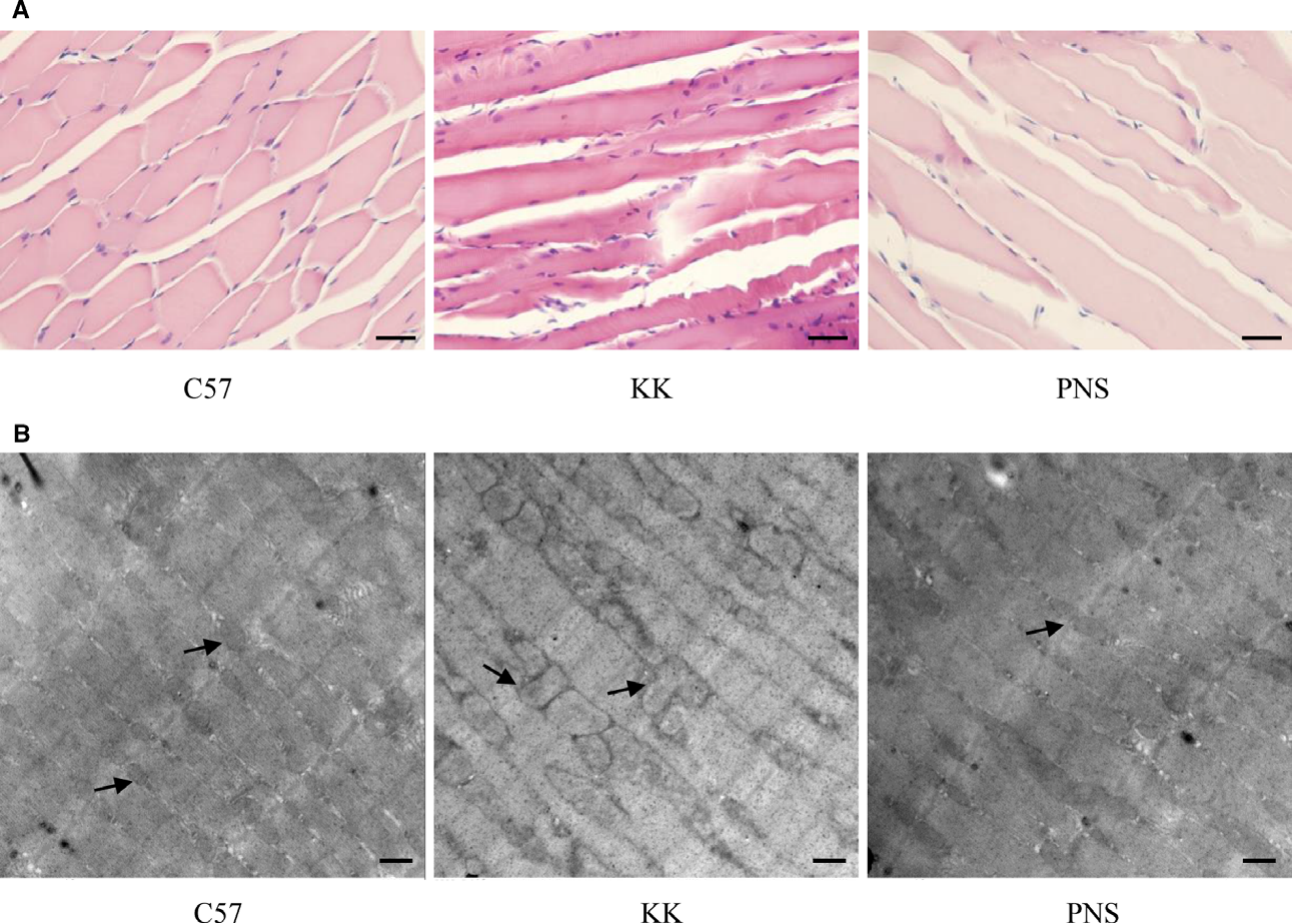
PNS‐mediated attenuation of diabetes‐induced damage in KKAy mouse skeletal muscle. (A) HE staining of skeletal muscle under the light microscope. Scale bar: 20 μm. (B) Skeletal muscle examined with transmission electron microscopy. Scale bar: 500 nm.

Pathological damage of skeletal muscle in KKAy mice was analyzed with HE‐stained sections (Fig. 2A). The skeletal muscle tissue arrangement in normal C57 mice was complete, regular and clear. However, in the diabetic KKAy mouse model, the skeletal muscle was abnormal and uneven. The cross‐striation of muscle became fuzzy. We also observed inflammatory cell infiltration and increased nuclear shifts. Following PNS treatment (200 μg·kg^−1^) for 6 weeks, the muscle cell arrangement became more complete and clear than that of KK group. Besides, inflammatory cell infiltration and small nuclear shifts were decreased.

We further analyzed the effect of PNS on ultrastructural changes using transmission electron microscopy (Fig. 2B). Skeletal muscle in the C57 group displayed sarcomere integrity, neatly arranged filaments, clear muscle line formation, and clear mitochondria boundaries. The double membrane structure of the mitochondria was visible (left panel of Fig. 2B). Conversely, the skeletal muscle fibers in the KKAy group were irregularly arranged, broken, dissolved, and unorganized. The mitochondria in the KKAy mice were swollen and deformed, the mitochondrial boundaries were blurred and irregular, and the structure of the bilayer plasma membrane was not visible (middle panel of Fig. 2B). After PNS treatment, the mitochondria were neatly arranged, and some areas of the double plasma membrane structure were visible. These finding, combined with the histology results, indicate that PNS treatment attenuates skeletal muscle damage caused by diabetes *in vivo*.

### Anti‐apoptotic properties of PNS in skeletal muscle from KKAy mice

**Figure 3:**
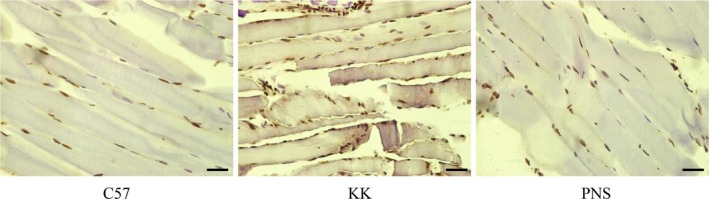
Anti‐apoptotic property of PNS in KKAy mouse skeletal muscle. TUNEL staining of skeletal muscles of C57BL/6J (left), KKAy (middle), and PNS‐treated KKAy mice (right). Scale bar: 20 μm.

Skeletal muscle apoptosis and atrophy can be seen in type II diabetes caused by stress signaling activation 16. We used TUNEL staining to investigate whether PNS attenuates diabetes‐induced skeletal muscle apoptosis (Fig. 3). TUNEL staining in the skeletal muscle from the C57 group was light blue‐brown and partially blue. A significant increase in TUNEL‐positive cells of skeletal muscle was observed in the KK group compared with the C57 group. PNS attenuated skeletal muscle apoptosis with fewer TUNEL‐positive cells.

### PNS attenuate high glucose‐induced down‐regulation of IRS1–PI3K–AKT signaling and GLUT4 expression in C2C12 cells

**Figure 4:**
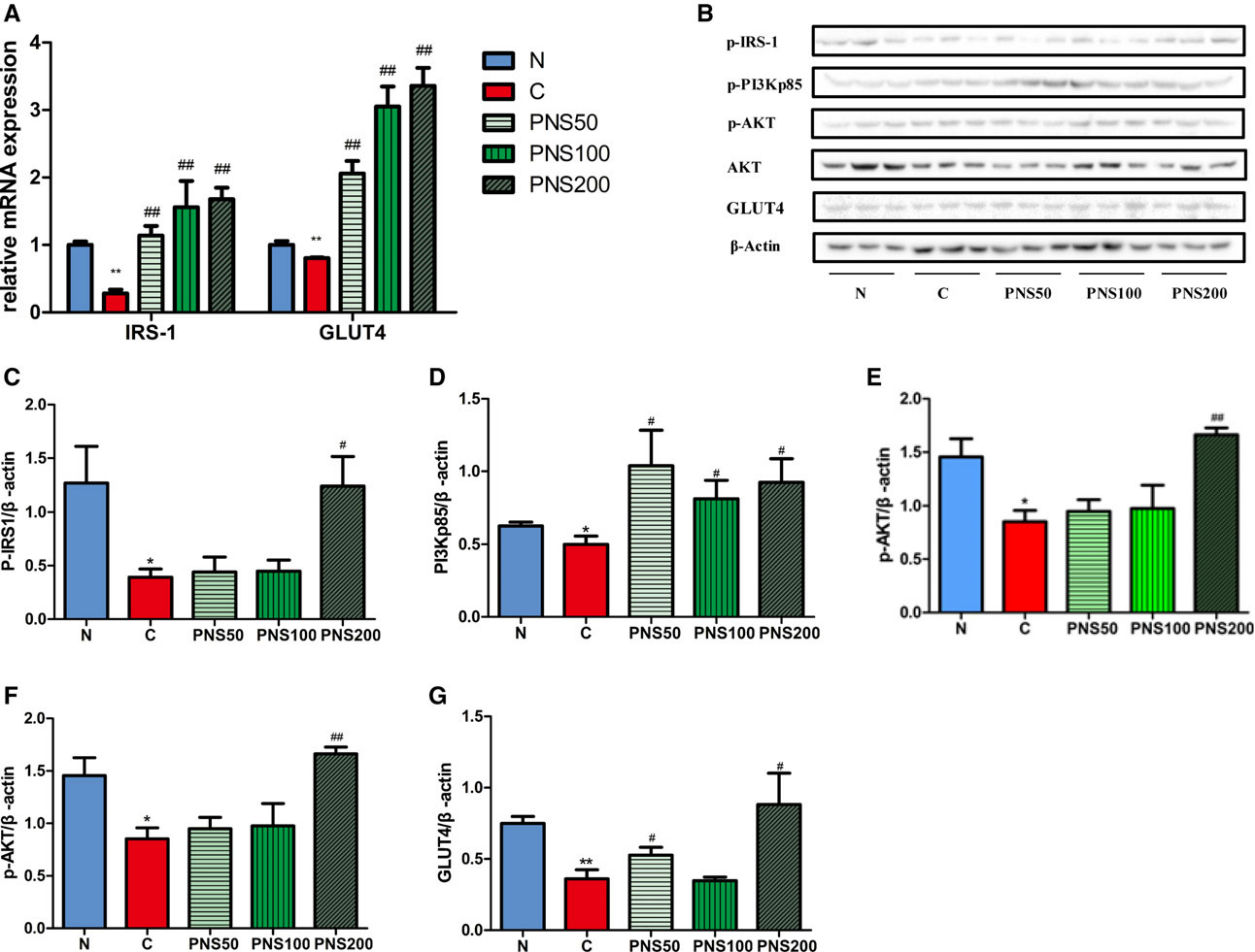
Effect of PNS on IRS1, PI3K, AKT and GLUT4 expression in C2C12 cells. High glucose medium‐incubated cells were used as positive controls (C) and complete medium‐incubated cells in low‐glucose were used as negative controls (N). (A) IRS1 and GLUT4 mRNA expression were analyzed. (B) Phosphorylated (p)‐IRS1, p‐PI3K (p‐PI3Kp85), and p‐AKT, as well as total AKT, GLUT4, and β‐actin were analyzed by western blot. (C–G) The relative expression levels of p‐Tyr612‐IRS1 (C), p‐PI3Kp85 (D), p‐AKT (E), AKT (F), and GLUT4 (G) were quantified by densitometry analysis and normalized to β‐actin. The results are expressed as the mean ± SD. *P < 0.05 vs. N group, **P < 0.01 vs. N group, ^#^ *P* < 0.05 vs. C group, ^##^ *P* < 0.01 vs. C group (Tukey's test, *n* = 3).

GLUT4 protein is an insulin‐regulated glucose transporter regulated by the PI3K–AKT signaling pathway. To evaluate the mechanisms of PNS treatment of diabetes, we analyzed the effects of PNS on PI3K–AKT signaling and GLUT4 expression in C2C12 cells cultured in high‐glucose (4.5 g·L^−1^) medium and treated with 50, 100, or 200 μg·mL^−1^ PNS, and in low‐glucose (1 g·L^−1^) cultured cells. IRS1 and GLUT4 mRNA measured by real‐time PCR were decreased by high glucose treatment (*P *<* *0.01). PNS treatment significantly up‐regulated mRNA expression of IRS1 and GLUT4 in a dose‐dependent manner (*P *<* *0.01) (Fig. 4A). Protein expression was detected by western blot of activated IRS1 (p‐Tyr612‐IRS1), PI3K (p‐PI3Kp85), and AKT (p‐AKT) as well as total AKT and GLUT4 (Fig. 4B). β‐Actin expression was used to normalize changes in protein expression. The protein expression of p‐Tyr612‐IRS1, p‐PI3Kp85, p‐AKT and GLUT4 were significantly down‐regulated in the high glucose treatment C group compared with low glucose N group, but increased in the C2C12 cells after PNS treatment (Fig. 4B–G). However, PNS treatment at 200 μg·mL^−1^ elicited the maximal response of p‐IRS1, p‐AKT and GLUT4. PI3K was sensitive to all three concentrations. Moreover, total AKT expression was decreased in the high glucose group and increased by PNS but not statistically significant (Fig. 4B,F).

### PNS attenuate the suppression of IRSI‐PI3K‐AKT signaling and GLUT4 expression in skeletal muscle from KKAy mice

**Figure 5:**
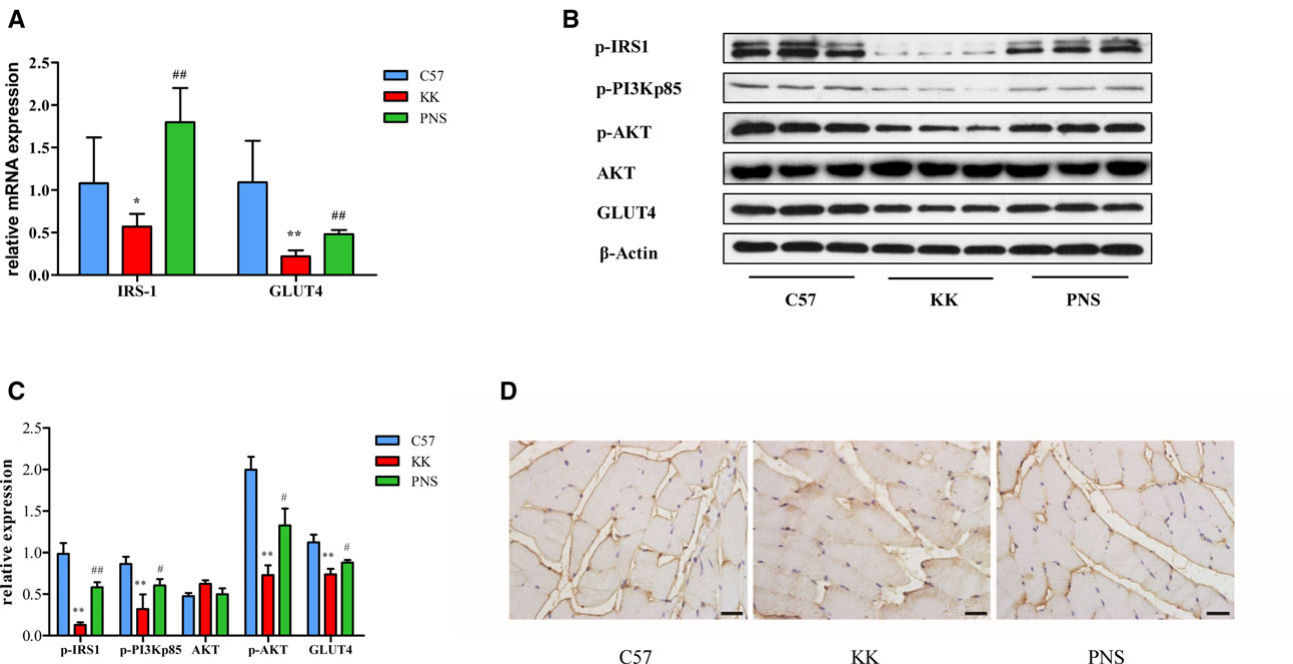
Effect of PNS on p‐IRS1, p‐PI3K, p‐AKT and GLUT4 in skeletal muscle from KKAy mouse. (A) Gene expression levels of IRS1 and GLUT4 were analyzed by real‐time PCR. (B,C) The protein expression level of p‐IRS1, p‐PI3Kp85, AKT, p‐AKT and GLUT4 were detected by western blot (B), quantified by densitometry analysis and normalized to β‐actin, with p‐AKT normalized to AKT (C). The results are expressed as the mean ± SD. *P < 0.05 vs. C57 group, **P < 0.01 vs. C57 group, ^#^ *P* < 0.05 vs. KK group, ^##^ *P* < 0.01 vs. KK group (Tukey's test, *n* = 3). (D) GLUT4 expression was further analyzed by immunohistochemical staining in skeletal muscles. Scale bar: 20 μm.

As shown in Fig. 5A, the skeletal muscle mRNA expression of IRS1 and GLUT4 was significantly decreased in the KKAy mice compared to the C57BL/6J mice. PNS recovered these gene expression levels (*P *<* *0.01). These alterations were further confirmed at the protein level by western blot (Fig. 5B,C); PNS prevented the diabetes‐induced down‐regulation of the expression of the IRS1–PI3K–AKT signaling pathway components p‐IRS1, p‐PI3Kp85, p‐AKT and GLUT4, while the total AKT remained unchanged. GLUT4 expression was further analyzed by immunohistochemical staining (Fig. 5D). In the C57 group, GLUT4 expression was mainly distributed in the cellular membrane. However, GLUT4 was scattered in both membrane and cytoplasm in the KKAy mice, but was primarily detected in the cytoplasm compared to the C57 mice. After PNS treatment, GLUT4 was still detected in both membrane and cytoplasm, but was primarily located in the cell membrane. These data indicate that PNS attenuate diabetes‐induced abnormal distribution of GLUT4 by activating the IRS1–PI3K–AKT pathway in diabetic skeletal muscle.

## Discussion

In this study, we investigated the anti‐diabetic effects of PNS in C2C12 and KKAy diabetic mice. PNS are the major anti‐diabetic component of *Panax notoginseng*, which has been used to treat diabetes and cardiovascular diseases for thousands of years in China 8, 17, 18. With increasing interest targeted on the use of natural products to treat diseases, PNS have been identified as a safe and effective candidate and recent studies have achieved promising results. PNS had an anti‐hyperglycemic, anti‐obesity and anti‐inflammatory effect and prevented kidney pathological changes in a diabetic mouse model and cell line 10, 19, 20, 21. In this study, we used the C2C12 cell line and KKAy mice as a model. The C2C12 cell line is a good model to explore the effects of medicine on glucose metabolism and insulin resistance 22. It has been reported that several herbal extracts, such as cinnamon water‐soluble extract, could increase the expression of GLUT4 in C2C12 cells 23. Another study used C2C12 cells to illustrate that palmitate contributes to insulin resistance through inhibiting phosphorylation of AKT in C2C12 myotubes 24. Our *in vitro* study has shown that PNS improved insulin‐mediated glucose uptake in a dose‐dependent manner. In fact, the safety of PNS was verified by a cell viability assay in C2C12 cells, which demonstrated that PNS did not affect skeletal muscle cell proliferation.

To future explore the effect and mechanism of PNS *in vivo*, the KKAy mouse was adopted as a type 2 DM model to investigate the effects of PNS on glucose metabolism, insulin resistance and pathological damage in skeletal muscle. The significantly increased FBG, RBG and HOMA‐IR as well as impaired glucose tolerance and insulin tolerance confirmed a stable diabetic and insulin‐resistant state of KKAy mice compared with C57BL/6J mice. Thus, they have been especially used to evaluate anti‐diabetic and anti‐obesity agents 25, 26. PNS diminished the diabetes‐induced elevation of FBG, RBG, ITT, OGTT, HOMA‐IR, and hyperlipidemia, which is consistence with other studies 9, 19. These results showed significant improvements in glucose tolerance and insulin sensitivity after treatment with PNS, combined with a reduction of TC and LDL. The beneficial effects of PNS on glucose tolerance and insulin tolerance indicate an improvement of whole body insulin sensitivity. Insulin resistance directly disturbs the physiological regulation of insulin in target organs, resulting in hyperlipidemia and hyperglycemia associated with diabetes, which in turn, feeds back on pancreatic islets and reduces insulin secretion 7. The importance of skeletal muscle in glucose metabolism is evidence by the observation that skeletal muscle consumes 80% of glucose during euglycemic hyperinsulinemic clamps 27. Impaired insulin signaling and abnormal metabolic pathways, as well as affecting muscle mass, mitochondrial damage, and oxidative stress in skeletal muscle, are commonly seen in DM 28. Although many studies have shown that PNS improve insulin resistance, few studies have defined the role of PNS on skeletal muscle in insulin resistance. Further studies on the mechanism of PNS regulation of insulin resistance were carried out to validate our hypothesis.

The PI3K–AKT signaling pathway is a classic vital pathway in muscle glucose metabolism. Insulin promotes glucose uptake through a series of signaling events initiated by insulin binding to insulin receptor and activating IRS1 phosphorylation. PI3K interacts with p‐IRS with subunit p85 to activate/phosphorylate its downstream effector, AKT. This response results in the cellular membrane translocation of GLUT4 to facilitate glucose uptake. Glucose is transported into skeletal muscle cells by GLUT4, which is mainly distributed in cell vesicles. Abnormal GLUT4 translocation can lead to glucose utilization disorders, resulting in insulin resistance 16, 29, 30. As GLUT4 translocation was increased by PNS treatment in muscle in our study and in 3T3‐L1 adipocytes reported by Kim *et al*. 10, we evaluated the expression of IRS1, PI3K and AKT phosphorylation since they are major upstream regulators of GLUT4 translocation. Here we described that PNS significantly increased IRS1 and GLUT4 gene expression in differentiated C2C12 cells and skeletal muscle from KKAy mice while western blot analysis confirmed these alterations at the protein level and showed that GLUT4, p‐AKT, p‐PI3Kp85, and p‐IRS1 expression was increased after PNS treatment in C2C12 cells and skeletal muscle from KKAy mice.

Our findings indicated that PNS increased GLUT4 expression and translocation to the plasma membrane via up‐regulating the phosphorylation of IRS1, AKT and PI3K in skeletal muscle tissue.

In spite of the fact that the antidiabetic effect of PNS has been studied, little attention has been paid to histological or mitochondrial ultrastructural change. Emerging evidence has indicated that the skeletal muscles in the type 2 diabetic KKAy mouse model are disordered, uneven, mildly atrophied, inflamed and displayed nuclear shifting and inflammatory cell infiltration. Observed with transmission electron microscopy, the skeletal muscle fibers in the KKAy mice presented as irregularly arranged, broken, dissolved, deformed and structurally unorganized and contained enlarged, deformed mitochondria 28, 31. In our study, mitochondrial damage and skeletal muscle damage were observed in the KK diabetic model group. PNS decreased these pathological alterations. The functions of mitochondria involve energy production, cellular signaling, and apoptosis. An imbalance in energy metabolism caused by mitochondrial dysfunction is a crucial factor in insulin resistance 10, 32. Even though the relationship between skeletal muscle mitochondria and the development of insulin resistance is still debatable, it is apparent that mitochondrial deficiencies are the cause of impaired fatty acid oxidation capacity and skeletal muscle fat accumulation, which lead to insulin resistance. Damaged mitochondrial boundaries were blurred and irregular, and the structure of the bilayer plasma membranes was fuzzy 10, 33, 34, 35. The same changes were observed in KKAy mice skeletal muscle but were reversed by PNS. In addition, TUNEL analysis of skeletal muscle from KKAy mice revealed that apoptotic injury contributes to skeletal muscle damage, consistent with Sishi's study 36. Interestingly, PNS treatment significantly attenuated this damage. From these results, we revealed that PNS could attenuate diabetes‐induced skeletal muscle damage, protect mitochondria and ameliorate myofibril damage.

## Conclusion

In conclusion, PNS possess anti‐diabetic properties, which improve glucose metabolism and insulin resistance, as evidenced by decreased FBG, RBG, ITT, OGTT, and HOMA‐IR. PNS also attenuated diabetes‐induced histopathological alterations and apoptotic damage in skeletal muscle. Furthermore, the down‐regulated IRSI–PI3K–AKT signaling pathway and GLUT4 expression were restored by PNS treatment. Our results demonstrate that PNS activate the IRSI–PI3K–AKT signaling pathway and up‐regulate GLUT4 expression to improve insulin resistance and rescues diabetic muscle pathological injury.

## Conflict of interest

The authors declare no conflict of interest.

## Author contributions

Conceived and designed the experiments: XuG, WS, GL and TL. Performed the experiments: XuG, WS, GX and DH. Analyzed the data: XuG, WS, GX, DH, LW, YH, XiG and XM. Contributed reagents/materials/analysis tools: GX, DH and LQ. Wrote the manuscript: XuG, WS, DH and TL.

## Supporting information


**Fig. S1.** C2C12 cell viability was measured and presented by absorbance at 450 nm. *n* = 10.
