# Supplementary material for: Panax notoginseng saponins alleviate skeletal muscle insulin resistance by regulating the IRS1–PI3K–AKT signaling pathway and GLUT4 expression
Source: FEBS Open Bio. 2019 Apr 26;9(5):1008–19. doi: 10.1002/2211-5463.12635 (PMC6487711; doi:10.1002/2211-5463.12635)
Supplement: Supplementary file 1 — Fig. S1. C2C12 cell viability was measured and presented by absorbance at 450 nm. n = 10. [file FEB4-9-1008-s001.docx]

Supplemental material for: **Panaxnotoginseng Saponins Alleviates Skeletal Muscle Insulin Resistance via Regulating IRS1-PI3K-AKT Signaling Pathway and GLUT4 Expression**

(Xuan Guo et al.)

**Supplementary Figure 1**


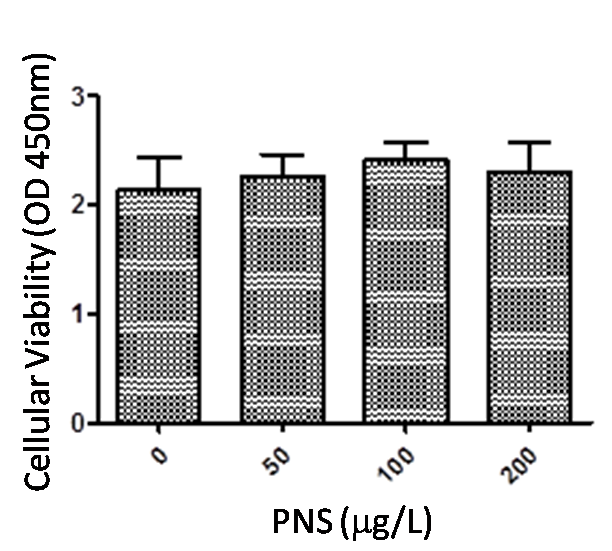


C2C12 cell viability was measured and presented by optical density at 450 nm (OD 450 nm). n= 10.
